# Post-Graduation Study

**Published:** 1905-09-02

**Authors:** 


### Post-Graduation Study.

In describing the arrangements and plans at present existing for the provision of medical education and training the announcements of the post-graduation colleges can by no possibility be omitted. Unknown, at least in this country, until a few years ago, these institutions have now reached a position of considerable prominence, and no one can doubt that they have a guarantee of permanence. This, in the first place, is indicated by their success. When it is intimated by each of the two leading post-graduation colleges in London that during the year several hundreds of practitioners have been in attendance at clinical and practical classes, it is manifest that these institutions are meeting a real and substantial demand. Further, there is every reason to anticipate that, so far from diminishing, this demand will increase and extend. At first, it is not unreasonable to suppose, the post-graduation movement was supported by a number of practitioners whose chief ambition was an increase of knowledge and a desire for efficiency, more particularly in the more recent methods of diagnosis and treatment, these ends in themselves being sufficient to justify self-denial and effort. Medicine has never lacked enthusiasts who find their chief reward in work for the work's sake, and the number of these is probably on the increase. But it is becoming increasingly evident that post-graduation study has a commercial as well as a scientific value. The public to a greater and greater extent appreciates efficiency, and demands from its medical advisers in ever-increasing measure the latest developments and applications of professional knowledge and skill. The practitioner who can supply these has a prospect much more sure than that of the man who is content with the older traditions. Thus on the mere question of practical business success, and apart from the gratification of professional enthusiasm, post-graduation opportunities are making a more and more confident appeal. That all this is greatly to the advantage of the public is obvious. In addition, it is equally to the advantage of the profession, as it contains a promise of larger opportunities for service in the public interest, where, in the last resort, the individual practitioner and the profession generally must find their justification and warrant. There is another possible relationship of post-graduation work which is not without interest. It is notorious that until comparatively recent years the education of the medical student has not comprised a practical training in the more special branches of practice, whilst it is equally certain that, during the same period, these departments of work have undergone enormous developments. One consequence of all this is that large numbers of medical practitioners have found themselves unable to deal with affections of the eye, ear, skin, etc., etc., and have been compelled to refer their patients in these departments to experts, even when in essence the condition was one of great simplicity. The fees expected, by no means unreasonably, by such experts are what many persons in the lower and middle classes are unwilling or unable to pay, and, as a result, these persons in large numbers are found in the clinics of the special hospitals. Everybody allows that this is an unsound economic position, yet under present conditions it is almost inevitable. The remedy may be expected in an increased and practical knowledge of the special departments on the part of the general practitioner. It is certain that many of the persons above alluded to would much prefer the payment of a moderate fee to attendance at the out-patient department, could they, for such payment, secure efficient attention. Here, it may be confidently stated, is the chance for the general practitioner, and more particularly for the younger man not yet over-burdened with work. Let him, whilst by no means disdaining the claims of general practice, select one or more departments in which to cultivate a more special and particular knowledge, and there is open for him a prospect full of promise. By such a policy he may gain not only the confidence of the public but also of a certain number of his senior and more busy *confrères.* As a mere business recommendation the cultivation of some special branch of practice should be urged on every practitioner, and its adoption in not a few instances has been a benefit both to the practitioner himself and also to his professional brethren and the entire neighbourhood. It provides not only the pleasure of good work and the practical rewards which follow this, but it is a help to the discouragement of the hospital habit which is so pernicious in its influence alike on the medical profession and the general public. The argument just stated is without doubt receiving an enlarged attention, and its stimulus may be traced in the increasing number of practitioners who take a temporary absence from their practice for the purpose of post-graduation study. For many reasons the majority of these find their way to London, and the opportunities there provided are both well-arranged and economical. The two principal institutions are the West London Hospital and College, and the Medical Graduates' College and Polyclinic.

### The West London Hospital and Post-Graduate College.

The West London Hospital is a fully equipped general hospital having 159 beds and an out-patient department at which some 30,000 new patients are seen every year. In addition to the general physicians and general surgeons the staff includes representatives of all branches of special practice, each of whom conducts an appropriate clinical service at the hospital. The whole of this practice, both indoor and outdoor, is utilised for purposes of clinical teaching. The college in connection with the hospital has been gradually extended and developed so that it may now claim to be a thoroughly well organised and well-equipped institution; recently there has been added a new pathological laboratory, and the arrangements for teaching microscopy and bacteriology have been brought well up-to-date. Lectures on medicine, surgery, etc., are given daily by members of the staff, and, for those who desire it, special instruction is provided with a view to prepare candidates for the higher examinations. There is ample provision for the convenience of practitioners attending the College in the shape of reading-room, smoking-room, library, etc., and though Hammersmith, where the hospital is situated, is not exactly in the centre of London, the facilities for getting there will satisfy the most exacting. The fees for hospital practice, including all the ordinary demonstrations and lectures, are £2 2s. for one month, £5 5s. for three months, £8 8s. for six months, and £12 12s. for 12 months. To put the position in brief, it is certain that for these fees the practitioner has every chance of following under experienced teachers the practice of a fairly large modern hospital. Any further information can be obtained on application to the Dean, Mr. L. A. Bidwell.

### The Medical Graduates' College and Polyclinic.

This institution offers many advantages for very modest fees. There is a special clinical service on five days of the week at 4 p.m., when selected cases are exhibited and made the text for a clinical commentary and discussion; and at 5.15 p.m., on four days of the week, there is also a clinical lecture or lantern demonstration. Both clinics and lectures are conducted by physicians and surgeons from the metropolitan schools and hospitals, so that they are often of special interest and value. The opportunities for seeing clinical cases and gathering the teaching they convey are, therefore, excellent. The fee for admission to this part of the work is £1 1s., and this includes the use of the museum, reading-room, etc. There are also practical classes for instruction in the use of the ophthalmoscope and laryngoscope, the estimation of refraction and the examination of cases of nervous disease, and in gynæcology, otology, bacteriology, etc. Special tutorial classes meet the needs of practitioners preparing for the higher examinations in medicine and surgery. A prospectus, with particulars of fees, etc., can be obtained from the Medical Superintendent, the Polyclinic, 22 Chenies Street, Gower Street, W.C.

### Charing Cross, Westminster, and Tottenham Hospitals.

Besides the above, arrangements for the instruction of medical practitioners are provided at Charing Cross, Westminster, and Tottenham Hospitals.

### The London Post-Graduate Association.

In addition, the "London Post-Graduate Association" issues tickets which admit to the clinical practice of a number of the leading general and special hospitals in the metropolis. The fee for a term of three months is £5 5s. Applications should be addressed to the Secretary, Examination Hall, Victoria Embankment, W.C.

